# The World’s Columbian Dental Congress

**Published:** 1892-04

**Authors:** 


### The World's Columbian Dental Congress.

At the annual meeting of the Southern Dental Association held in Atlanta, July, 1890, action was taken looking to the organization of a great meeting of the dental profession to be held in Chicago, in 1893, and after due deliberation it was decided to appoint from that body five persons to act in conjunction with a like number that might be appointed by the American Dental Association. At the meeting of the latter body at Excelsior Springs in August following, the action of the Southern Association was approved, their recommendation adopted, and five from that body were appointed who should co-operate with the committee above referred to. These two committees were authorized to add to their number from one to five persons, as they might see fit. This afterwards resulted in the selection of five persons, additional, making the Executive Committee to consist of fifteen persons. This committee so constituted was given full power to take such action as in its judgment it may deem best for creating an organization for the purpose of holding a Dental Congress in Chicago, in 1893, which the reputable dentists throughout the world shall be invited to attend, and that any action that this committee may take in the premises shall be final and binding.

The following named persons constitute this general Executive Committee, viz.: From the Southern: L. D. Carpenter, Atlanta, Ga.; J. Y. Crawford, Nashville, Tenn.; W. J. Barton, Paris, Texas; J. Taft, Cincinnati, Ohio; and C. S. Stockton, Newark, N. J. From the American: L. D. Shepard, Boston, Mass.; W. W. Walker, New York City; A. O. Hunt, Iowa City, Iowa; H. B. Noble, Washington, D. C.; and Geo. W. McElhaney, Columbus, Ga.

Then selected by these: J. C. Storey, Dallas, Texas; M. W. Foster, Baltimore, Md.; A. W. Harlan and John S. Marshall, of Chicago, Ills.; and H. J. McKellops, St. Louis, Mo.

This General Executive Committee has held a number of meetings for the purpose of arranging the work of this Congress, which has resulted thus far in the appointment of twenty-four committees, who are to have in charge the preparation and carrying out of the various branches or departments of the work of the Congress. Below will be found a list of these committees:

This list of committees will give some idea of the intended scope of the work. Doubtless other committees will be added as the work developes.

### COMMITTEES AS APPOINTED AND CONFIRMED TO DATE.

### GENERAL EXECUTIVE COMMITTEE.

Chairman—Dr W W Walker, 67 W. 9th St., N. Y. City; Secretary—Dr A O Hunt, Iowa City, Iowa; Treasurer—Dr John S Marshall, 9 Jackson St., Chicago, Ill.; Dr W J Barton, Paris, Texas; Dr L D Carpenter, Atlanta, Ga.; Dr J Y Crawford, Nashville, Tenn.; Dr M W Foster, 9 Franklin St., Baltimore, Md.; Dr A W Harlan, 70 Dearborn St., Chicago, Ill.; Dr H J McKellops, 2630 Washington Ave., St. Louis, Mo.; Dr G W McElhaney, Columbus, Ga.; Dr H B Noble, N. Y. Ave., Washington, D. C.; Dr John C Storey, Dallas, Texas; Dr C S Stockton, Newark, N, J.; Dr L D Shepard, 330 Dartmouth St., Boston, Mass.; Dr J Taft, 7th St., Cincinnati, Ohio.

COMMITTEE OF CONFERENCE FOR WORLD'S CONGRESS AUXILIARY.

W D Miller, Berlin, Germany; F Busch, Berlin, Germany; Thos W Evans, Paris, France; E Magitot, Paris, France; G W Sparrock, Lima, Peru; W B Macleod, Edinburg, Scotland; A W W Baker, Dublin, Ireland; Ernest Sjoberg, Stockholm, Sweden; Charles S. Tomes, London, England; W H Coffin, London, England; W Geo Beers, Montreal, Canada; H C Edwards, Madrid, Spain; E Lecaudy, Paris, France; J G Van-Martin, Rome, Italy; Plattschick, Pavia, Italy; Joseph Arkovy, Buda Pesth, Hungary; C Redard, Geneva, Switzerland; W H Morgan, Nashville, Tenn.; W H Dwinelle, New York City; R B Winder, Baltimore, Md.; Elisha G Tucker, Boston, Mass.; W W H Thackston, Sarmville, Va.; J B Rich, Washington, D. C.; J D White, Philadelphia, Pa.: W H Eames, St. Louis, Mo.; J B Patrick, Charleston, S. C.; F J S Gorgas, Baltimore, Md.; G V Black, Jacksonville, Ill. ; R Finlay Hunt, Washington, D. C.; E Bacon, Portland, Maine; Benjamin Lord, New York City, N. Y.; A L Northrop, New York City; W W Allport, Chicago, Ill.; W W Walker, New York City; L D Carpenter, Atlanta, Ga.; J Y Crawford, Nashville, Tenn.; W J Barton, Paris, Tex.; J Taft, Cincinnati, O.; C S Stockton, Newark, N.J.; L D Shepard, Boston, Mass.; H J McKellops, St. Louis, Mo.; A O Hunt, Iowa City, Iowa; H B Noble, Washington, D.C.; Geo W McElhaney, Columbus, Ga.; J C Storey, Dallas, Tex.; M W Foster, Baltimore, Md.; A W Harlan, Chicago, Ill.; J S Marshall, Chicago, Ill.

---

Committee No. 1. GENERAL FINANCE COMMITTEE.

Chairman—L D Shepard, 330 Dartmouth St., Boston, Mass.; T W Brophy, 96 State St., Chicago, Ill.; A L Northrop, New York City.

Committee No. 2.

PROGRAMME COMMITTEE—NOT APPOINTED.

Committee No. 3. COMMITTEE ON EXHIBITS.

Chairman—Charles Pruyn, 70 Dearborn St., Chicago, Ill.; Arthur E Matteson, 3700 Cottage Grove Ave., Chicago, Ill.; E M S Fernandez, 103 State St., Chicago, Ill.

Committee No. 4.

COMMITTEE ON TRANSPORTATION.

Chairman—F H Gardiner, 126 State St., Chicago; V H Jackson, 240 Lenox Avenue, New York City; Geo Eubank, Birmingham, Ala.

Committee No. 5. COMMITTEE ON RECEPTION.

Chairman—W W Allport, 9 Jackson St., Chicago; W W H Thackston, Farmville, Va.; E M S Fernandez, 103 State St., Chicago; Geo A Christmann, Staats Zeitung Building, Chicago; Jas McManus, 32 Pratt St., Hartford, Conn.; Elisha G Tucker, Boston, Mass.; J D Thomas, 912 Walnut St., Philadelphia, Pa.; H J McKellops, 2630 Washington Av., St. Louis; L L Dunbar, 500 Sutter St., San Francisco, Cal.; V E Turner, Raleigh, N. C.; Joseph Bauer, 130 Esplanade St., New Orleans, La.; J F P Hudson, 19 West 39th St., New York City; W P Dickinson, 608½ Nicollett Ave., Minneapolis, Minn.; C F W Holbrook, 34 Park St., Newark, N. J.; W J Foster, 9 West Franklin St., Baltimore, Md.; R M Sanger, East Orange, N. J.

Committee No. 6.

COMMITTEE ON REGISTRATION.

Chairman—Fred A Levy, 343 Main St., Orange, New Jersey; E L Clifford, 401 West Monroe St., Chicago, Ill.; Geo N West, 34 Monroe St., Chicago, Ill.; J Y Crawford, Nashville, Tenn.; C V Rosser, Atlanta, Ga.; T L James, Fairfield, Iowa; W H Fundenburgh, 323 Pennsylvania Avenue, Pittsburg.

Committee No. 7.

COMMITTEE ON PRINTING TRANSACTIONS—NOT APPOINTED.

Committee No. 8.

COMMITTEE ON CONFERENCE WITH STATE AND LOCAL SOCIETIES.

Chairman—J Taft, Cincinnati, Ohio.

LIST OF STATE COMMITTEES.

*Alabama*—E S Chisholm, Tuscaloosa, Chairman; A Eubank, Birmingham; Chas P Robinson, Mobile; G M Rousseau, Montgomery.

*Alaska*————————

*Arizona*—L N Goodrich, Phœnix, Chairman; D Pentland, Prescott; J Hardy, Phœnix; W Warnekross, Tombstone.

*Arkansas*—M·C Marshall, Little Rock, Chairman; W B Pollard, Hot Springs; L K Land, Pine Bluff; R D Seals, Fort Smith; A E Kimmons, Fort Smith.

*California*—C L Goddard, San Francisco, Chairman; W J Younger, San Francisco; E L Townsend, Los Angeles.

*Colorado*—P T Smith, Denver, Chairman; W E Griswold, Denver; H P Kelly, Denver; R B Weiser, Georgetown.

*Connecticut*—E S Gaylord, New Haven, Chairman; Jas McManus, Hartford; R W Browne, New London.

*Delaware*—T H Gilpin, Middleton, Chairman; C R Jefferis, Wilmington.

*District of Columbia*—Henry C Thompson, Washington, Chairman; R B Donaldson; J Hall Lewis; L C F Hugo; H M Schooley.

*Florida*—J N Jones, Jacksonville, Chairman; James Chace, Ocala; Duff Post, Tampa; I J Welch, Pensacola.

*Georgia*—S B Barfield, Macon, Chairman; John H Coyle, Thomasville; H H Johnson, Macon; W C Wardlaw, Augusta.

*Idaho*—E L P Ector, Moscow, Chairman; John H McCallie, Moscow; A Boston, Lewiston.

*Illinois*—W H Taggart, Freeport, Chairman; C N Johnson, Chicago; J J Jennelle, Cairo.

*Indiana*—J B Morrison, Indianapolis, Chairman; P G C Hunt, Indianapolis; S B Browne, Fort Wayne.

*Indian Territory*——————

*Iowa*—C B Peterson, Dubuque, Chairman; S C Hatch, Sioux City; L K Fullerton, Waterloo.

*Kansas*—L C Wasson, Topeka, Chairman; C E Esterley, Lawrence; Wm H Schulze, Atchison.

*Kentucky*—C G Edwards, Louisville, Chairman; Chas E Dunn, Louisville; F Peabody, Louisville.

*Louisiana*—C E Kells, Jr., New Orleans, Chairman; Joseph Bauer, New Orleans; Andrew G Friederichs, New Orleans.

*Maine*—D W Fellows, Portland, Chairman; Edmund C. Bryant, Pittsfield; Henry A Kelly, Portland.

*Maryland*—E P Keech, Baltimore, Chairman; A J Volck, Baltimore; Edward Nelson, Frederick.

*Massachusetts*—D M Clapp, Boston, Chairman; W H Potter, Secretary; Eugene H Smith, Boston; L D Shepard, Boston; S G Stevens, Boston; D B Ingalls, Clinton; R R Andrews, Cambridge; Geo A Maxfield, Holyoke; James H Daly, Boston.

*Michigan*—C S Case, Jackson, Chairman; Geo L Field, Detroit; F L Owen, Grand Rapids.

*Minnesota*—T E Weeks, Minneapolis, Chairman; M G. Jenison, Minneapolis; C H Robison, Wabasha.

*Mississippi*—Morgan Adams, Sardis, Chairman; R K Luckie, Holly Springs; J D Miles, Vicksburg; G B Clements, Macon.

*Missouri*—C L Hungerford, Kansas City, Chairman; A H Fuller, St. Louis; J D Patterson, Kansas City.

*Montana*—C S Whitney, Miles City.

*Nebraska*—H T King, Fremont, Chairman; A W Nason, Omaha; H C Miller, Grand Island; H J. Cole, Norfolk; I W Funck, Beatrice.

*Nevada*—A Chapman, Virginia City, Chairman; M A Greenlaw, Reno; S S Southworth, Carson City.

*New Hampshire*—C W Clements, Manchester, Chairman; G A Young, Concord; Wm Jarvis, Claremont; W R Blackstone, Manchester; C H Hayward, Peterborough; B C Russell, Keene.

*New Jersey*—S C G Watkins, Mont Clair, Chairman; B F Luckey, Paterson; R M Sanger, E. Orange; Charles A Meaker, Newark.

*New Mexico*————

*New York*—John I Hart, New York City, Chairman; K C Gibson, New York: W Carr, New York; M L Chaim, New York; Chas Butler, Buffalo; F.A Remington, New York.

*North Carolina*—V E Turner, Raleigh, Chairman; J H Durham, Wilmington; J F Griffith, Salisbury.

*North Dakota*—S J Hill, Fargo, Chairman; S P Johnson, Grand Forks; W O DePuy, Bismarck; H S Sowles, Wahpeton; E M Pierce, Hillsboro.

*Ohio*—D R Jennings, Cleveland, Chairman; H F Harvey, Cleveland; M H Fletcher, Cincinnati; L E Custer, Dayton; A F Emminger, Columbus.

*Oklahoma Territory*—D A Peoples, Guthrie, Chairman; G F Dean, Oklahoma City; J S Nickolson, El Reno.

*Oregan*—S J Barber, Portland, Chairman; E G Clark, Portland; J R Cardwell, Portland.

*Pennsylvania*—L A Faught, Philadelphia, Chairman; C S Beck, Wilkesbarre; J A Libbey, Pittsburg.

*Rhode Island*————

*South Carolina*—Thos T Moore, Columbia, Chairman; W S Brown, Charleston; A P Johnstone, Anderson; B H Teague, Aiken.

*South Dakota*—O M Huestis, Aberdeen, Chairman; C W Stutenroth, Watertown; F W Blomily, Sioux Falls.

*Tennessee*—H W Morgan, Nashville, Chairman; B S Byrnes, Memphis; W H Richards, Knoxville; H E Beach, Clarksville.

*Texas*—W R Clifton, Waco, Chairman; G M Patton, Galveston; Tom Robinson, Houston; Geo S Staples, Sherman; T L Westerfield, Dallas; H J McBride, Tyler.

*Utah*—A S Chapman, Salt Lake City, Chairman: A B Dunford, Salt Lake; F W Baker, Ogden.

*Vermont*—G F Cheney, St. Johnsbury, Chairman; Thomas Mound, Rutland; R M Chase, Bethel.

*Virginia*—J Hall Moore, Richmond, Chairman; W W H Thackston, Farmville; Jos R Woodley, Norfolk; E P Beadles, Danville; T H Parramore, Hampton; D W Rust, Alexander.

*Washington*—W E Burkhardt, Tacoma, Chairman; F P Hicks, Tacoma; J C Grasse, Seattle.

*West Virginia*—H H Harrison, Wheeling, Chairman; Jno H McClure, Wheeling; H K Jones, Parkersburg; George I Keener, Grafton; J N Mahan, Charleston.

*Wisconsin*—B G Marcklein, Milwaukee, Chairman; C C Chittenden, Madison; George H McCausey, Janesville.

*Wyoming*—Waiting for nominations.

Committee No. 9.

COMMITTEE ON THE HISTORY OF DENTAL LEGISLATION IN THIS AND OTHER COUNTRIES.

Chairman—William Carr, New York City, N. Y.; Paul Dubois, 2 Rue d'Amsterdam, Paris; F Busch, Berlin, Germany; J H Mummery, London, England; M Etcheparaborda, Buenos Ayres, South America.

Committee No. 10. AUDITING COMMITTEE.

Chairman—L D Shepard, Boston, Mass.; R R Andrews, Cambridge, Mass.; Chas A Meeker, Newark, N. J.

Committee No. 11. COMMITTEE ON INVITATION.

Chairman—W C Barrett, 208 Franklin St., Buffalo, N. Y.; E T Darby, 1513 Walnut St., Philadelphia, Pa.; S G Perry 46 West 37th St., New York City; W C Wardlaw, Augusta, Ga.; S W Dennis, 81 Flood Building, San Francisco, Cal.; J D Patterson, Kansas City, Mo.

Committee No. 12.

COMMITTEE ON MEMBERSHIP.

Chairman—Edmund Noyes, 65 Randolph St., Chicago, Ill.; B F Luckey, Paterson, N. J.; E S Chisholm, Tuscaloosa, Alabama; C M Bailey, 28 Syndicate Block, Minneapolis, Minn.; Dan'l N McQuillen, 1628 Chestnut St., Philadelphia, Pa.

Committee No. 13.

COMMITTEE ON EDUCATION AND LITERARY EXHIBITS.

Chairman—J J R Patrick, Belleville, Ill.; J Y Crawford,

Nashville, Tenn.; A H Fuller, 2602 Locust St., St. Louis, Mo.; C A Brackett, 102 Truro St , Newport, R. I.; B H Catching, Atlanta, Ga.

Committee No. 14.

COMMITTEE ON CLINICS IN OPERATIVE DENTISTRY AND ORAL SURGERY.

Chairman—C F W Bodecker, 60 East 58 St., New York City; S C G Watkins, Montclair, N. J.; John S Marshall, 9 Jackson St., Chicago, Ill.; Arthur B Freeman, 325 West Madison St., Chicago, Ill.; H H Schumann, 240 Wabash Ave., Chicago, Ill.; Henry W Morgan, Nashville, Tenn.; William Crenshaw, Atlanta, Ga.

Committee No. 15.

COMMITTEE ON PROSTHETIC DENTISTRY.

Chairman—S H Guilford, Philadelphia, Pa.; L P Haskell, 211 Wabash Ave., Chicago, Ill.; A P Johnstone, Anderson, South Carolina; W N Morrison, St. Louis, Mo.; Fred C Barlow, 646 Jersey Ave., Jersey City; J Hall Lewis, 1309 F St., N. W., Washington, D. C.; A O Hunt, Iowa City, Iowa; R R Freeman, Nashville, Tenn.; E S Gaylord, New Haven, Conn.

Committee No. 16.

LOCAL COMMITTEE OF ARRANGEMENTS.

Chairman—E S Talbot, 125 State St., Chicago, Ill.; F H Gardiner, 126 State St., Chicago, Ill.; C N Johnson, Opera House Building. Chicago, Ill.; D B Freeman, 4000 Drexel Boulevard, Chicago, Ill.; H J McKellops, 2630 Washington Ave., St. Louis, Mo.

Committee No. 17. COMMITTEE ON ESSAYS.

Chairman—E C Kirk, Philadelphia, Pa.; J W Wassall, Chicago, Ill.; A H Thompson, Topeka, Kansas; H H Johnson, 26 2d St., Macon, Ga.; L G Noel, Nashville, Tenn.

Committee No. 18.

COMMITTEE ON HISTORY OF DENTISTRY IN THE UNITED STATES.

Chairman—J Taft, Cincinnati, Ohio; Louis Jack, 1315 Locust St., Philadelphia, Pa.; F T VanWort, Brooklyn, N. Y.; F J S Gorgas, Baltimore, Md.; H L McKellops, 632 Sutter St., San Francisco, Cal.; E G Betty, Cincinnati, Ohio; J B Patrick, Charleston, South Carolina.

Committee No. 19.

ON NOMENCLATURE—NOT APPOINTED.

Committee No. 20.

COMMITTEE TO PROMOTE THE APPOINTMENT OF DENTAL SURGEONS IN THE ARMIES AND NAVIES OF THE WORLD.

Chairman—M W Foster, Baltimore; B Holly Smith, Baltimore; Geo Cunningham, Cambridge, England; De Gallippe, Paris; Adolph Weil, Munich; Jno E Gievers, Amsterdam, Holland; E DeTrey, Vevey, Switzerland; A Szigmondy, Vienna; O Mela, Geneva, Italy; V Haderup, Copenhagen; O J Chrustchow, St. Petersburg, Russia; Alex McG Denham, Monjitas 68½, Chili; Geo B Newland, 107 Calle Florida, Buenos Ayres.

Committee No. 21.

COMMITTEE ON CARE OF THE TEETH OF THE POOR.

Chairman—W J Barton, Paris, Texas; C A Brackett, Newport, R. I.; T D Ingersoll, Erie, Pa.: W M Fisher, Dundee, Scotland.

Committee No. 22.

COMMITTEE ON BIOLOGY AND BACTERIOLOGY.

Chairman—R R Andrews, Cambridge, Mass.; M H Fletcher, Cincinnati, Ohio; W X Sudduth, Minneapolis, Minn.; W D Miller, Berlin, Germany; J H Mummery, London, England; D E Caush, Brighton, England; E Magitot, Paris, France; M Morgensten, Baden Baden, Germany; Geo S Allan, New York City, N. Y.

Committee No. 23. COMMITTEE ON PRIZE ESSAYS.

Chairman—Theo Stanley, Kansas City, Mo.; C S Stockton, Newark, N. J.

Committee No. 24. EDITORIAL COMMITTEE.

Chairman—W W Walker, New York City; A O Hunt, Iowa City; L D Shepard, Boston; J Taft, Cincinnati; J S Marshall, Chicago.

Committee No. 25. NOMINATING COMMITTEE.

Chairman—W W Walker, New York City; A W Harlan, Chicago, Ill.; John S. Marshall, Chicago, Ill.

